# Supplementary figures and images for: Phage cocktail strategies for the suppression of a pathogen in a cross‐feeding coculture
Source: Microb Biotechnol. 2020 Aug 19;13(6):1997–2007. doi: 10.1111/1751-7915.13650 (PMC7533344; doi:10.1111/1751-7915.13650)

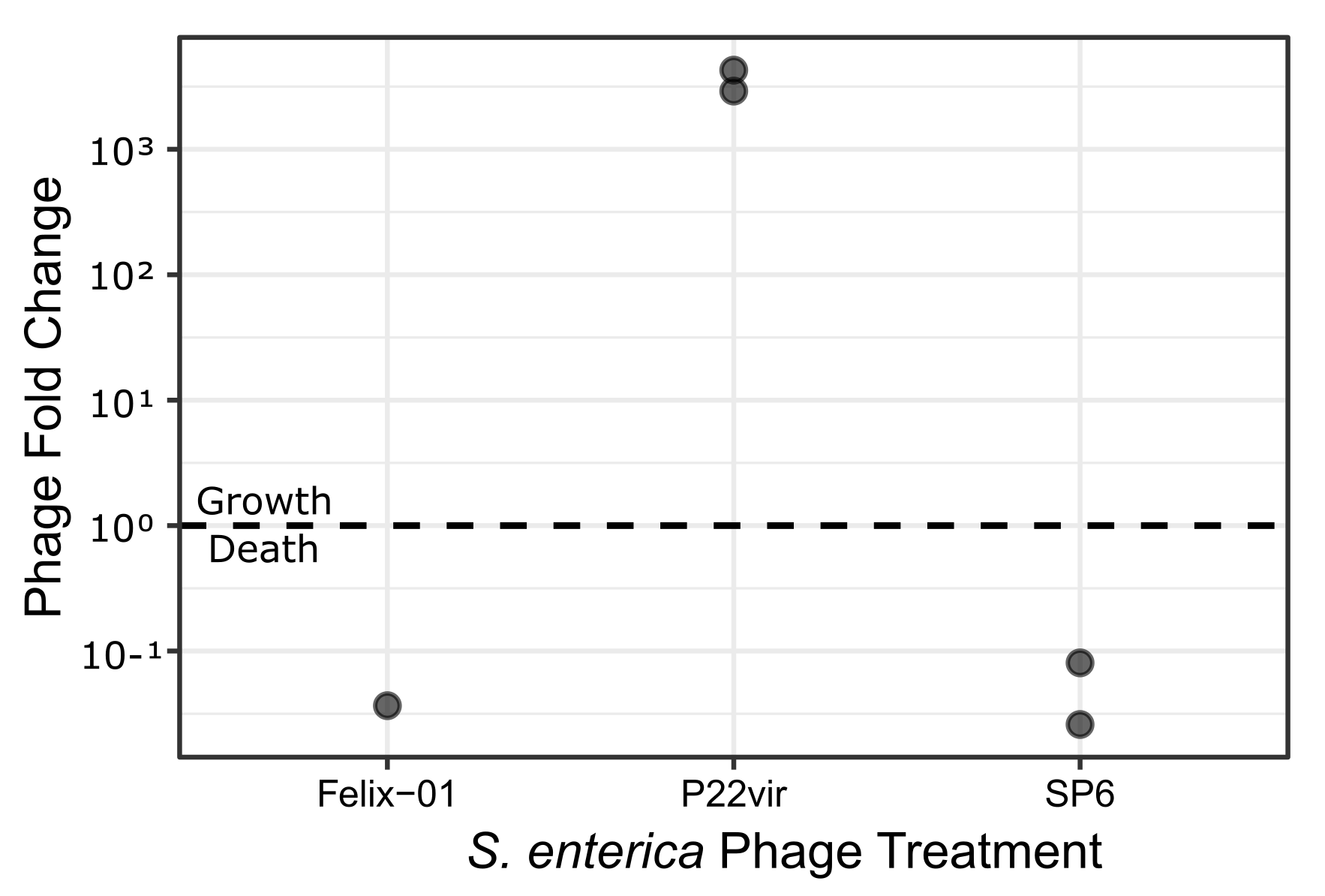

Supplement: Supplementary file 1 — Fig. S1. Screening of S. enterica‐specific phage activity in cooperative coculture. P22vir, SP6, and Felix‐01 S. enterica‐specific phages were inoculated into E. coli‐S. enterica cocultures and grown at 30°C while shaking until stationary phase was reached (4–5 days, n = 1–2). Initial and final PFU ml−1 were measured by plating with ancestral S. enterica. Only P22vir increased in concentration over the growth period. [file MBT2-13-1997-s001.png]

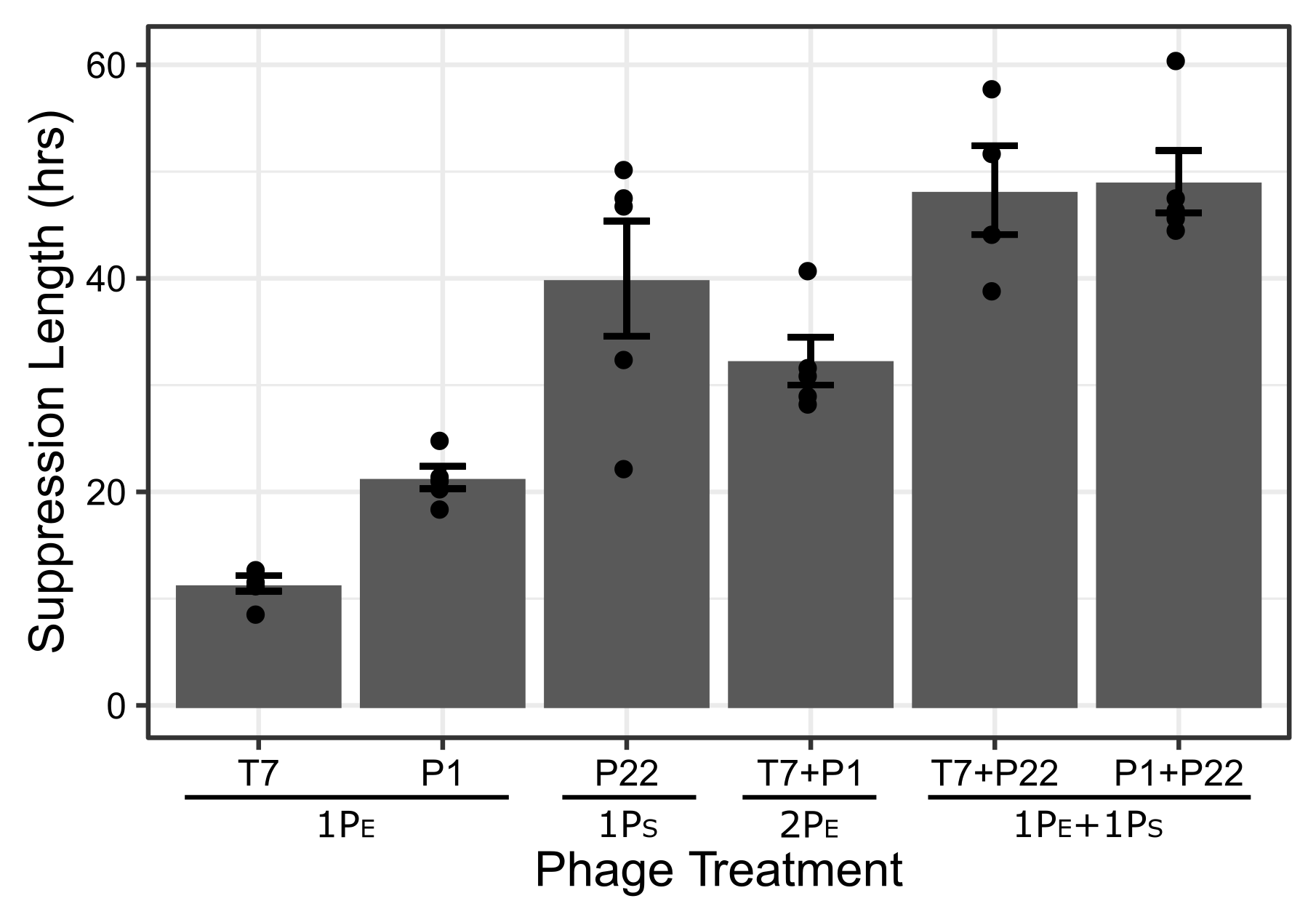

Supplement: Supplementary file 2 — Fig. S2. Coculture‐level suppression lengths caused by phage treatments. Relative coculture suppression lengths of single and cocktail phage treatments standardized to the no phage control. Suppression length was calculated using 95% maximum OD600. Bars represent means ± SE (n = 4–5). [file MBT2-13-1997-s002.png]

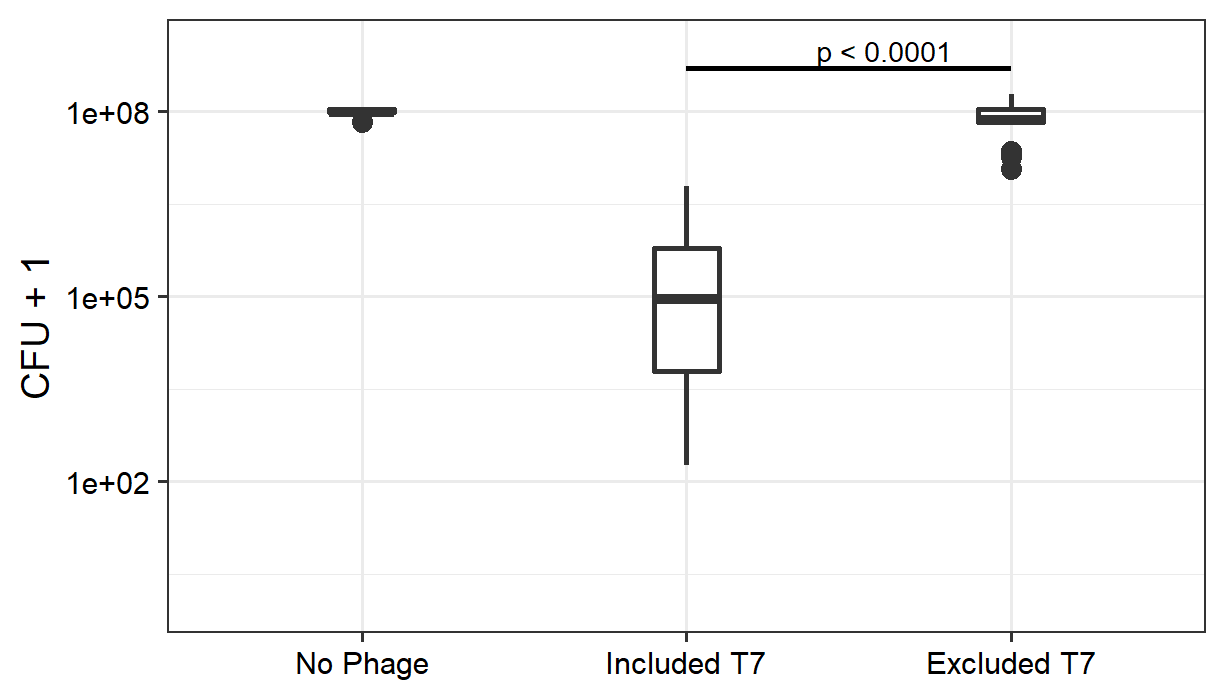

Supplement: Supplementary file 3 — Fig. S3. Boxplots of final E. coli densities after phage treatments. Including T7 phage in treatments lowered final E. coli population size. Cocultures were grown with single phage treatments and cocktails and bacterial populations sizes were counted by plating with selective plates. Statistical significance was tested with a Two‐sample Mann–Whitney U. (n = 15). [file MBT2-13-1997-s003.png]
