# Supplementary material for: Phage cocktail strategies for the suppression of a pathogen in a cross‐feeding coculture
Source: Microb Biotechnol. 2020 Aug 19;13(6):1997–2007. doi: 10.1111/1751-7915.13650 (PMC7533344; doi:10.1111/1751-7915.13650)
Supplement: Supplementary file 4 — Table S1. Absolute and relative suppression lengths of phage treatments. Table S2. Parameters for resource‐explicit ODE mathematical model. Table S3. Starting densities of cross‐ and dual‐resistance modeling. [file MBT2-13-1997-s004.docx]

**Supporting Figures & Tables**

| 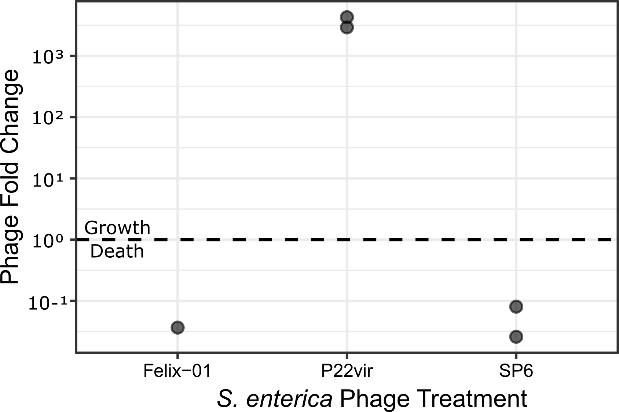 |
| --- |
| **Supplemental Figure 1. Screening of *S. enterica*-specific phage activity in cooperative coculture.** P22*vir*, SP6, and Felix-01 *S. enterica*-specific phages were inoculated into *E. coli-S. enterica* cocultures and grown at 30°C while shaking until stationary phase was reached (4-5 days, n = 1-2). Initial and final PFU/ml were measured by plating with ancestral *S. enterica.* Only P22*vir* increased in concentration over the growth period. |

| 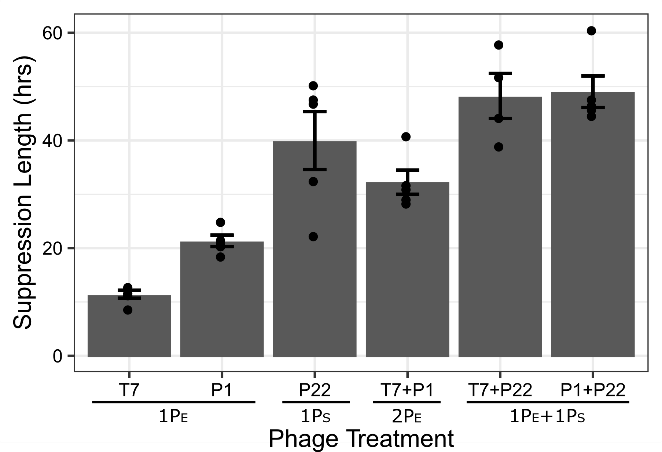 |
| --- |
| **Supplemental Figure 2. Coculture-level suppression lengths caused by phage treatments.** Relative coculture suppression lengths of single and cocktail phage treatments standardized to the no phage control. Suppression length was calculated using 95% maximum OD600. Bars represent means ± SE (n = 4-5). |

**
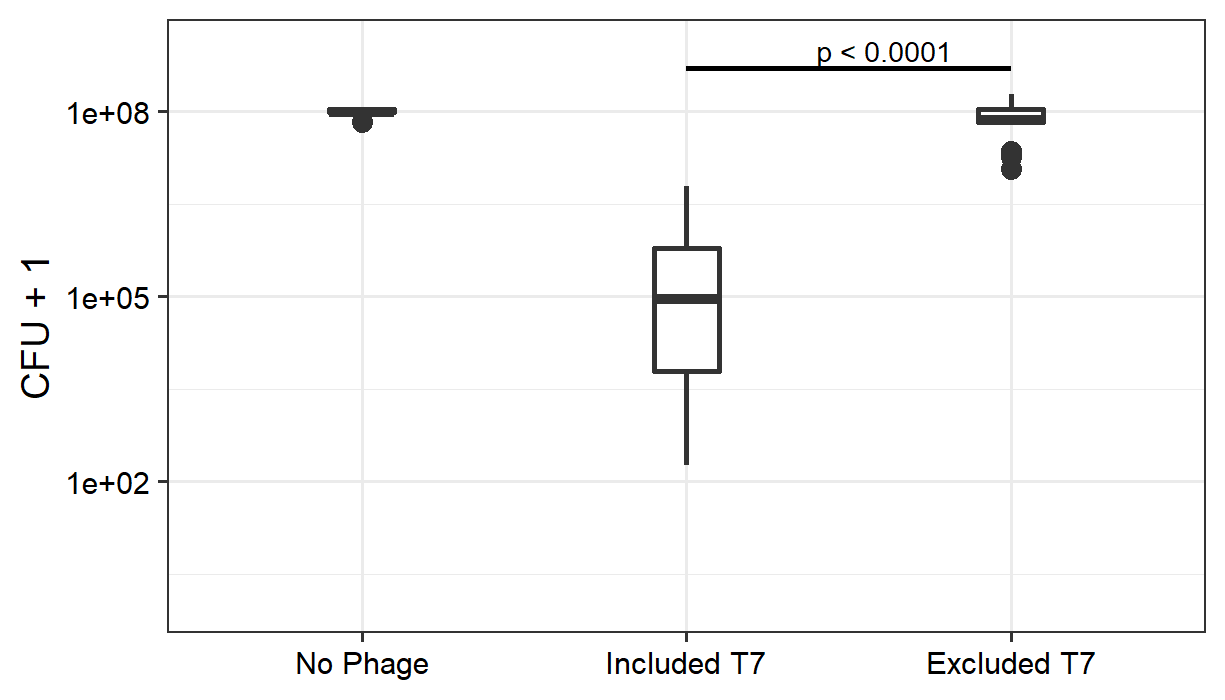
**

**Supplemental Figure 3. Boxplots of final *E. coli* densities after phage treatments.** Including T7 phage in treatments lowered final *E. coli* population size. Cocultures were grown with single phage treatments and cocktails and bacterial populations sizes were counted by plating with selective plates. Statistical significance was tested with a Two-sample Mann–Whitney U. (n = 15)

**Table S1. Absolute and relative suppression lengths of phage treatments.**

| **Phage Treatment** | **Treatment Type** | **No. Replicate Communities** | **Absolute Suppression Length**  **(hrs ± SE)** | **Relative Suppression Length (standardized to phage-free)** |
| --- | --- | --- | --- | --- |
| Phage-free | None | 5 | 34.44 ± 0.0 | 1.00 ± 0.0 |
| T7 | Single phage  (1E) | 5 | 49.74 ± 2.05 | 1.44 ± 0.06 |
| P1vir | Single phage  (1E) | 5 | 58.37 ± 2.35 | 1.69 ± 0.07 |
| P22vir | Single phage  (1S) | 5 | 77.7 ± 12.0 | 2.26 ± 0.35 |
| T7+P1vir | Pathogen-targeting  (2E) | 5 | 67.98 ± 3.28 | 1.97 ± 0.10 |
| T7+P22vir | Multispecies-targeting  (1E+1S) | 4 | 84.03 ± 10.42 | 2.44 ± 0.30 |
| P1vir + P22vir | Multispecies-targeting  (1E+1S) | 5 | 86.3 ± 6.56 | 2.5 ± 0.19 |

**Table S2. Parameters for resource-explicit ODE mathematical model.**

| **Parameter (name in model)** | **Parameter value** |
| --- | --- |
| *E. coli* growth rate (mu_e) | 0.291/hr |
| *S. enterica* growth rate (mu_s) | 0.221/hr |
| *E. coli* production of ace (p_e_ace) | 4e-12 grams produced/*E. coli* cell |
| *S. enterica* consumption of ace (c_s_ace) | 3e-12 grams consumed/*S. enterica* cell |
| *S. enterica* production of met (p_s_met) | 4e-12 grams produced/*S. enterica* cell |
| *E. coli* consumption of met (c_s_met) | 3e-12 grams consumed/*E. coli* cell |
| *E. coli*-specific T7 burst size (burst_T7) | 100 phage/burst *E. coli* cell |
| *E. coli*-specific T7 adsorption rate | 1e-9 /phage**E. coli* cell |
| *E. coli*-specific P1*vir* burst size (burst_P1) | 100 phage/burst *E. coli* cell |
| *E. coli*-specific P1*vir* adsorption rate | 1e-9 /phage**E. coli* cell |
| *S. enterica*-specific P22*vir* burst size (burst_P22) | 100 phage/burst *S. enterica* cell |
| *S. enterica*-specific P22*vir* adsorption rate | 1e-9 /phage**E. coli* cell |

**Table S3. Starting densities of cross- and dual-resistance modeling.**

| Simulated Organism | Organism Description | Number of Cells | |
| --- | --- | --- | --- |
|  |  | Cross-Resistance | Dual-Resistance |
| Es | Sensitive *E. coli* | 9.98999 X 10^5^ | 9.97999 x 10^5^ |
| ErT7 | T7-resistance *E.coli* | 0 | 10^3^ |
| ErP1vir | P1vir-resistant *E. coli* | 0 | 10^3^ |
| Er | T7- and P1vir-resistant *E. coli* | 1.001 x 10^3^ | 1 |
| Ss | Sensitive *S. enterica* | 9.98999 x 10^5^ | 9.97999 x 10^5^ |
| SrP22vir | P22vir-resistant *S. enterica* | 0 | 10^3^ |
| Sr2 | Phage2-resistant *S. enterica* ^a^ | 0 | 10^3^ |
| Sr | P22vir and Phage2-resistant  *S. enterica* | 1.001 x 10^3^ | 1 |
| Total Biomass | Sum of all bacterial biomass | 10^6^ | 10^6^ |

^a^ Phage 2 is for simulation purposes only and does not correspond to a second experimental *S. enterica* phage.
